# The Bone Extracellular Matrix as an Ideal Milieu for Cancer Cell Metastases

**DOI:** 10.3390/cancers11071020

**Published:** 2019-07-20

**Authors:** Alexus D. Kolb, Karen M. Bussard

**Affiliations:** Department of Cancer Biology, Thomas Jefferson University, Philadelphia, PA 19107, USA

**Keywords:** bone extracellular matrix, breast cancer, prostate cancer, multiple myeloma, metastasis, bone remodeling, mechanotransduction

## Abstract

Bone is a preferential site for cancer metastases, including multiple myeloma, prostate, and breast cancers.The composition of bone, especially the extracellular matrix (ECM), make it an attractive site for cancer cell colonization and survival. The bone ECM is composed of living cells embedded within a matrix composed of both organic and inorganic components. Among the organic components, type I collagen provides the tensile strength of bone. Inorganic components, including hydroxyapatite crystals, are an integral component of bone and provide bone with its rigidity. Under normal circumstances, two of the main cell types in bone, the osteoblasts and osteoclasts, help to maintain bone homeostasis and remodeling through cellular communication and response to biophysical signals from the ECM. However, under pathological conditions, including osteoporosis and cancer, bone remodeling is dysregulated. Once in the bone matrix, disseminated tumor cells utilize normal products of bone remodeling, such as collagen type I, to fuel cancer cell proliferation and lesion outgrowth. Models to study the complex interactions between the bone matrix and metastatic cancer cells are limited. Advances in understanding the interactions between the bone ECM and bone metastatic cancer cells are necessary in order to both regulate and prevent metastatic cancer cell growth in bone.

## 1. Introduction

Bone is a unique organ that provides structural support for the body. The bone, including the extracellular matrix (ECM) is constantly remodeled where ECM components are degraded, modified, and secreted [1]. The balance between degradation and secretion of the bone ECM is important in maintaining bone density, elasticity, and strength [2]. The bone is composed of many different cell types, including, but not limited to epithelial cells, osteoblasts, osteoclasts, osteocytes, and fibroblasts [3,4]. These cells are surrounded by organic components, most notably collagen type I, and inorganic components, including hydroxyapatite crystals [3,4,5]. These components encompass the bone matrix which provide a complex network of biochemical and physiological cues that contribute to bone processes, including bone remodeling and mechanotransduction [4]. 

The bone is an attractive site for cancer colonization [6]. Normal bone processes, such as bone remodeling, are deregulated leading to disorganization of the bone matrix and abnormal behavior of cells [7]. Once in bone, cancer cells utilize normal products of bone remodeling, such as collagen type I, to fuel cancer growth [2,8]. Cancer cells recruit other cells in the microenvironment, such as fibroblasts or osteoblasts to remodel collagen I products, resulting in a disorganized and stiff matrix [2,8,9]. As a result, ECM dynamics, composition, and integrity are disrupted, ultimately altering the interactions of native bone cells with their microenvironment and promoting tumor invasion [8]. Understanding the interactions between the bone ECM and bone metastatic cancer cells are crucial in order to both regulate and prevent metastatic cancer cell growth in bone. 

## 2. Bone Physiology

**Figure 1:**
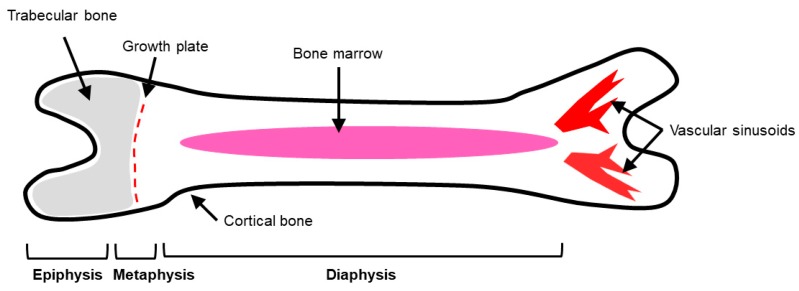
Gross anatomy of the long bones. Depicted are the three regions of long bones: epiphysis, metaphysis, and diaphysis. The outermost layer of bone is composed of densely packed cortical bone, while the interior and ends of bone are made up of trabecular bone (gray region). The growth plate is located in the metaphysis region and allows for longitudinal growth of bone. The bone marrow is located in the diaphysis, or shaft of long bones. Vascular sinusoids are located in the epiphysis of bone and allow for sluggish blood flow into the bone.

Bone is composed of two main types: cortical and trabecular bone (Figure 1) [10,11]. Cortical and trabecular bone have varying structural properties, which affect how each type responds to mechanical loading [12,13,14]. Differences between cortical and trabecular bone are determined by density and porosity [14,15]. For example, long bones, such as the femur, have three regions: the epiphysis, metaphysis, and diaphysis [16]. Long bones have an interior of porous, trabecular bone located in the epiphyses, and a hard exterior shell of cortical bone [13,16]. Located between the metaphysis and the diaphysis is the epiphyseal plate, or growth plate, which is a marker of longitudinal bone growth [5,10]. The diaphysis of the long bones contain the bone marrow surrounded by hard, cortical bone [16].

Cortical bone, otherwise known as compact bone, surrounds the exterior of bone [10,11]. Cortical bone is composed of densely packed collagen type I fibrils and is highly mineralized, in comparison to trabecular bone [10,11]. Therefore, cortical bone adds strength and rigidity to the skeleton [10,17]. The mechanical properties of cortical bone are characterized by the porosity, mineralization, and organization of the bone microstructure [13]. 

Cancer cells frequently disseminate to bone and utilize changes in the bone matrix to promote cancer growth [2,18,19]. For example, lysyl oxidase (LOX) secretion by cancer cells is upregulated at metastatic sites and contributes to tissue stiffness [20]. Tissue stiffness is a factor known to promote cancer colonization and outgrowth [2,8].

Trabecular bone, on the other hand, is a porous, loosely organized matrix compared to the compact structure of cortical bone [11]. Trabecular bone is located near the ends of bone just below the growth plate and is accompanied by red bone marrow, a hematopoietic tissue that produces red blood cells, and blood vessels, including sinusoids [10,11]. Sinusoids are large blood vessels that facilitate sluggish blood flow, permitting entry of cells from circulation into the bone space [21]. Trabecular bone provides flexibility to bone due to its porous structure, forming along lines of stress, ultimately acting as a shock absorber [10,17]. Due to its dynamic nature, trabecular bone undergoes remodeling more frequently compared to cortical bone, which also increases its metabolic activity [12,13]. Trabecular bone also differs in mechanical properties [13]. For example, trabecular bone is known to have decreased strength and stiffness compared to cortical bone, but other mechanical properties, such as elasticity and density are heterogeneous [13,15]. The heterogeneity of trabecular bone is also due to its high bone turnover rate, ultimately changing bone microstructure [12,13].

### 2.1. Bone Cells Modulate the Bone Microenvironment

**Figure 2:**
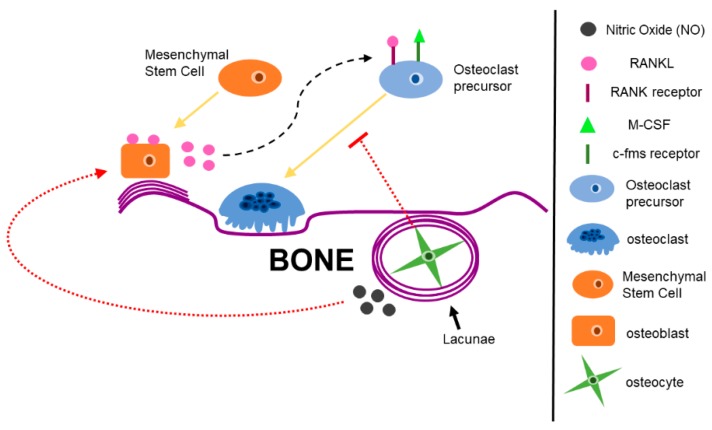
Osteoblasts, osteoclasts, and osteocytes are key modulators in the bone microenvironment. Osteoblasts derived from mesenchymal stem cells and are responsible for bone building. Osteoblasts have bound and soluble forms of receptor activator of nuclear-factor kappa-β ligand (RANKL), a key factor needed for osteoclast differentiation. Secreted RANKL can bind to the receptor activator of nuclear-factor kappa-β (RANK) receptor on osteoclast progenitor cells and initiate differentiation of osteoclast precursors. Osteoclasts are derived from a monocyte/macrophage lineage. Along with soluble RANKL, macrophage-colony stimulating factor (M-CSF) binds to the colony-stimulating factor-1 (C-FMS) receptor on osteoclast progenitors to initiate osteoclast differentiation. RANKL and M-CSF are the two key factors in osteoclast differentiation and formation. Once osteoclastogenesis is initiated, mono-nucleate osteoclast precursors fuse together to form multi-nucleate, mature osteoclasts. Osteocytes are terminally differentiated osteoblasts that have become embedded in the bone matrix. Osteocytes secrete factors in response to mechanical strain, including nitric oxide, which can activate osteoblast bone formation or inhibit osteoclast formation and bone resorption.

All sites of bone are metabolically active, which contributes to bone homeostasis and bone remodeling [10]. Bone homeostasis and remodeling is tightly controlled by cells of the bone microenvironment: bone-forming osteoblasts, bone-resorbing osteoclasts, and osteocytes (Figure 2) [10,22,23].

Osteoblasts, derived from mesenchymal stem cells, participate in bone mineralization, bone remodeling, and production of bone ECM proteins (Figure 2) [4,22]. Mature osteoblasts participate in bone mineralization and bone matrix protein production by secreting organic molecules, such as collagen type I, and inorganic molecules, such as proteoglycans, to form bone and bone matrix [4,22,24]. Matrix metalloproteinases (MMPs) are also secreted from osteoblasts to aid in matrix degradation [25]. Osteoblasts also express factors that initiate osteoclastogenesis, such as receptor activator of nuclear-factor kappa-β ligand (RANKL) and macrophage colony stimulating factor (M-CSF), the two cytokines needed for osteoclast differentiation [26,27]. Lastly, osteoblasts can become embedded in their own mineralized matrix [10]. Osteoblasts secrete type I collagen, which is eventually converted into a hard matrix by calcium phosphate where they become trapped and cannot divide [10]. These non-dividing and trapped osteoblasts are called osteocytes [10]. 

Osteocytes are terminally differentiated osteoblasts that reside in a small cavity called a lacunae (Figure 2) [10]. Osteocytes make up about 90–95% of all bone cells [22]. Osteocytes are able to interact with the bone microenvironment and surrounding cells through tiny channels called canaliculi connected to the lacunae [10]. Canaliculi include osteocyte processes which allow for interaction with other cells in the bone microenvironment, as well as allow for the exchange of waste and nutrients [28]. Osteocytes are known to have critical roles in regulating bone remodeling and sensing mechanical stimuli in bone [4,29,30,31]. One way osteocytes sense mechanical loading in bone is by fluid flow shear stress, which is a physical deformation in the bone matrix that causes load-induced changes in the flow of liquid through the canalicular network [28]. Osteocytes secrete factors in response to mechanical strain, including fluid flow shear stress [28]. These factors regulate osteoblast and osteoclast function [28]. Nitric oxide (NO) is one of the factors secrete by osteocytes under mechanical strain [28,32]. NO decreases osteoblast proliferation and increase osteoblast differentiation in vitro [32,33]. Osteocytes also regulate osteoclast activation during bone remodeling [28]. It has been proposed that osteocytes secrete osteoclast-inhibitors, such as osteoprotegerin, and only when a population of osteocytes die, does this secretion reverse, and osteoclasts become active [34]. In addition to bone remodeling, osteocytes are also critical in sensing mechanical load in bone, terming them the ‘mechanosensor of bone’ [35,36,37]. Osteocytes are also mechanotransducers, meaning they can convert mechanical stimuli into biological output, such as initiating a biochemical signaling pathway [28].

**Figure 3:**
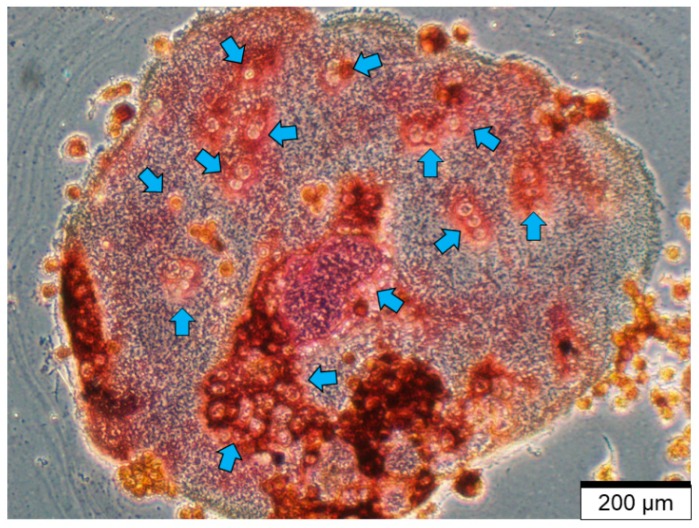
Mature, multi-nucleated osteoclast in vitro. Pictured is one mature osteoclast differentiated with exogenous RANKL. The osteoclast has multiple nuclei (blue arrows) and stained tartrate resistant acid phosphatase (TRAP) positive, indicative of a mature osteoclast. (pink-brown stain). Scale = 200 μm.

Osteoclasts are derived from hemopoietic stem cells in the bone marrow and are differentiated from the monocyte-macrophage lineage (Figure 2) [4,10,38]. RANKL and macrophage colony stimulating factor (M-CSF) are the two ligands needed for osteoclast progenitor differentiation [39,40,41]. Once M-CSF and RANKL are bound to their respective receptors, mononuclear osteoclast progenitor cells fuse with one another, eventually forming large, multi-nucleate osteoclasts (Figure 3) [10,38]. 

Mature osteoclasts are characterized by their large size, presence of many nuclei, and a ruffled border membrane [10,38,42,43]. Active osteoclasts are further characterized in vitro by (1) formation of a resorptive pit on dentin [44,45]; (2) formation of an actin ring [43] and; (3) number of multi-nucleated cells that stain positive for tartrate resistant acid phosphatase (TRAP) [42,46]. During bone resorption, osteoclasts express α_v_β_1_ integrin to interact with bone matrix through the tripeptide Arg-Gly-Asp (RGD) binding domains of non-collagenous proteins, such as osteopontin, bone sialoprotein, or fibronectin [47,48]. In this way, a sealing zone is formed, whereby the ruffled border membrane seals the site of degradation and releases a number of acids, to degrade bone minerals, and lysosomal enzymes, such as TRAP and cathepsin K [10,49]. These enzymes aid in degradation of organic components of the bone matrix [10,49]. When resorbing bone, osteoclasts release cytokines, hormones, and growth factors, including transforming growth factor-β (TGF-β) that are stored in the bone matrix [4]. Active TGF-β regulates both osteoclast bone resorption and osteoblast bone formation, whereby low concentrations of active TGF-β induces osteoclast precursors to migrate to bone resorptive pits [50,51]. In addition, active TGF-β released during bone resorption has been shown to stimulate bone mesenchymal stem cell (MSC) recruitment to sites of bone resorption [52].

### 2.2. Bone Remodeling During Homeostasis and Disease

**Figure 4:**
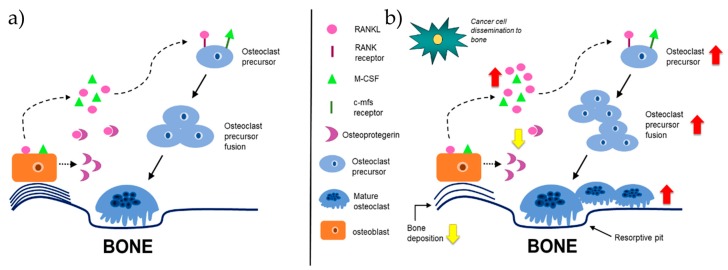
The bone matrix is manipulated to promote cancer growth. (**a**) The bone matrix is comprised of organic and inorganic components. The main organic component of the bone matrix is collagen type I. The bone matrix is also comprised of non-collagenous proteins, including fibronectin, lysyl oxidase (LOX), a crosslinker of collagen, and matrix metalloproteinases (MMPs), degraders of the bone matrix. Inorganic bone matrix components include hydroxyapatite crystals, which allow for mineral exchange in bone. To combat excess bone degradation, osteoblasts secrete osteoprotegerin (OPG), a decoy receptor for soluble receptor activator of nuclear-factor kappa-β ligand (RANKL). OPG will bind the soluble RANKL and inhibit it from binding to the RANK receptor on osteoclast progenitors. Debris from bone resorption is removed and osteoblast progenitors are recruited to the resorption site, where they differentiate into osteoblasts, and secrete bone-forming molecules. Osteoblasts secrete organic components, including collagen type I and non-collagenous proteins, including proteoglycans, that aid in new bone formation. In this way, bone homeostasis is maintained with little net gain or loss of bone composition. (**b**) Cancer cells initiate osteoblasts to secrete excess RANKL. Secreted RANKL binds to the RANK receptor on osteoclast progenitor cells to initiate differentiation. Increases in mono-nucleate osteoclast precursors cause more fusion and initiate increased production of multi-nucleate, mature osteoclasts. Due to increases in mature osteoclast formation, there is an increase in bone degradation. Additionally, OPG secretion decreases from osteoblasts. Therefore, there is less OPG to bind soluble RANKL, contributing to the indirect increase of osteoclast differentiation. The introduction of cancer cells into the bone microenvironment disrupts communication between osteoblasts and osteoclasts.

Bone remodeling is accomplished by both bone-forming osteoblasts and bone-resorbing osteoclasts (Figure 4a) [10,22]. First, the bone microenvironment sends an initiating signal to start bone remodeling [22]. Initiating remodeling signals include hormones, such as parathyroid hormone (PTH), or mechanical stimuli, such as fluid flow shear stress [4,22]. Osteoblasts respond to the mechanical stimuli generated by the osteocytes or to direct hormonal signals and recruit osteoclasts to the site of remodeling [22]. Osteoblasts secrete RANKL and macrophage-colony stimulating factor (M-CSF), the two factors that stimulate osteoclast resorption [22,26,27]. RANKL is expressed in two forms: a bound form and a secreted form [53]. Secreted RANKL will bind to the RANK receptor [42,53,54] and M-CSF will bind to the colony-stimulating factor-1 (c-Fms) receptor on osteoclast progenitor cells to promote osteoclast differentiation [4,22,42]. 

Once committed to osteoclast lineage, osteoclast progenitors fuse together to become large, multi-nucleate cells indicative of mature osteoclasts that resorb bone [42]. Mature, active osteoclasts bind to the Arg-Gly-Asp (RGD) binding site and the sealing zone is formed, whereby hydrolases and acids are secreted into the resorptive pit [10,42]. To combat excess bone degradation, osteoblasts secrete osteoprotegerin (OPG), a decoy receptor for soluble RANKL [55,56]. OPG binds the soluble RANKL and inhibits it from binding to the RANK receptor on osteoclast progenitors, therefore decreasing osteoclastogenesis (Figure 4a) [56]. Debris from bone resorption is removed and osteoblast progenitors are recruited to the resorption site, where they differentiate into osteoblasts, and secrete bone-forming molecules [22]. Osteoblasts secrete organic components, including collagen type I and non-collagenous proteins, including proteoglycans, that will form new bone [22]. In this way, bone homeostasis is maintained with little net gain or loss of bone composition [22,57].

Under pathological conditions, including osteomyelitis and bone metastatic cancers, bone remodeling is disrupted (Figure 4b) [58,59]. Bone metastatic cancer cells take advantage of and utilize the plethora of cytokines, chemokines, growth factors, and cell adhesion molecules that are released into the bone niche as a result of dysregulated bone remodeling [17]. This observation was originally described 130 years ago in 1889 by Stephen Paget [60]. Paget first recognized that the movement of cancer cells within the body was nonrandom and was also unexplained by blood flow: “When a plant goes to seed, its seeds are carried in all directions; but they can only grow if they fall on congenial soil.” [60]. As suggested by Paget, cancer cells are the “seeds” and the bone microenvironment, rich in growth factors, chemokines, and cytokines, is the “congenial soil” necessary for cancer cell growth [60]. Paget’s “seed and soil” hypothesis also explains the preferential metastasis of certain types of cancer cells, including breast, prostate, lung, and multiple myeloma, to the bone.

In bone metastatic cancers, and especially in those that result in osteolytic disease, there is an increase in osteoclast resorption and decrease in osteoblast bone formation, resulting in overall bone loss, with no new bone deposition [61]. The most well-known example of this is the ‘vicious cycle’ of breast cancer metastasis to bone [19,26,62]. Metastatic breast cancer cells hijack the process of bone remodeling by producing parathyroid hormone related protein (PTHrP), which stimulates osteoblasts to produce RANKL [19]. RANKL binds to the RANK receptor on osteoclast progenitors and stimulates osteoclast differentiation and bone resorption [19]. There is an increase in the release of cytokines, growth factors, and minerals from bone resorption by osteoclasts, further contributing to the ‘vicious cycle’ of bone degradation [19]. 

Overall, bone cells are responsible for many functions in the bone microenvironment, including but not limited to, bone remodeling; modulation of growth factors and cytokines; mechanosensing, and mechanotransduction [4,22,30,42]. The bone matrix is intertwined with bone remodeling, allowing for dynamic interactions between the organic and inorganic components of bone and matrix proteins [4,5,63]. Together, bone components and matrix proteins are able to facilitate bone homeostasis. 

## 3. The Bone Extracellular Matrix

**Figure 5:**
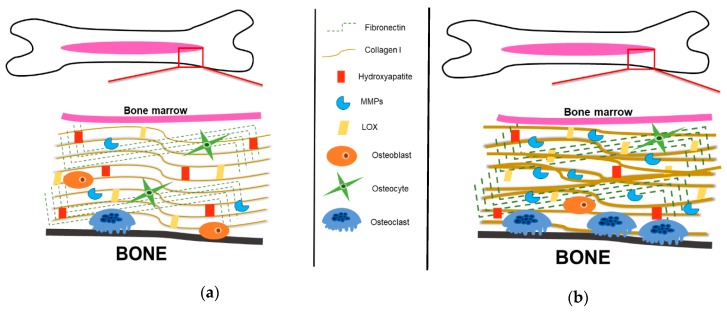
The bone matrix is manipulated to promote cancer growth. (**a**) The bone matrix is comprised of organic and inorganic components. The main organic component of the bone matrix is collagen type I. The bone matrix is also comprised of non-collagenous proteins, including fibronectin, lysyl oxidase (LOX), a crosslinker of collagen, and matrix metalloproteinases (MMPs), degraders of the bone matrix. Inorganic bone matrix components include hydroxyapatite crystals, which allow for mineral exchange in bone. (**b**) Under disease conditions, such as cancer, the bone matrix is constantly being remodeled. The biggest change is altered collagen production. Collagen fibrils become thick, dense, and unorganized compared to their linear, aligned counterparts. Fibronectin production increases, as well as LOX crosslinking, causing increases in tissue stiffness. MMP production also increases, leading to excess bone matrix degradation and remodeling.

The bone ECM is a dynamic structure that encompasses the organic and inorganic components of bone (Figure 5) [5]. The bone ECM contributes to many different cellular processes, including cell attachment, differentiation, and migration; tissue repair and regeneration; and structural and functional support of the tissue [4,5,49]. The bone matrix serves as the foundation for bone growth, repair, and cellular interactions [5,64,65]. The bone ECM is composed of both organic and inorganic components that contribute to the structure and function of the bone [4,5].

### 3.1. Organic Bone Components: Collagenous Proteins

Bone is composed predominantly of a fibril-forming collagen matrix, accounting for about 90% of the bone matrix [5]. Collagen is an important component of bone matrix because it provides strength and stability to the skeleton, as well as serves as a scaffold for bone formation, cell attachment, and utilized as a mechanical stimulus for biochemical signaling [4,66,67]. Collagen type I is the most abundant form of collagen in bone matrix [5,63,66,68]. Typical collagen type I fibers are triple helical structures, formed from two α1 chains and one α2 chain wound tightly together into a triple helix structure [5,63]. The triple helix structure is equivalent to one collagen molecule [10]. These collagen molecules then pack together to form the collagen fibrils that form the bone matrix [10]. Collagen fibers are organized in a hierarchical manner and arrange in a directional manner corresponding to cellular orientation [68,69]. The biochemical and biophysical properties of collagen type I fibers are known to affect cellular behaviors, such as cell proliferation, differentiation, and survival [70,71]. For example, cells respond differently to denatured collagen than mature, crosslinked collagen fibrils [72]. In addition, collagen fibers in bone are highly cross-linked under normal conditions, which makes the bone matrix insoluble [73], except during bone remodeling [22]. The insolubility of the bone matrix contributes to bone strength and stiffness [4,10,67]. Changes in collagen can also occur under pathological conditions, such as aging and cancer, which can cause bone weakness and fragility [5,74]. In bone metastatic cancers, type I collagen production and orientation is known to be altered [66,68]. There is increased collagen production at the bone metastatic site, whereby secreted collagen molecules are dense, misaligned, and disorganized, further disrupting bone mechanical function (Figure 5b) [66,75]. Liu et al. identified that metastatic breast cancer cells secreted miR-218 which directly regulated type I collagen secretion from osteoblasts in the bone niche [76]. The authors further identified elevated levels of miR-218 in blood samples from patients with breast cancer bone metastases, suggesting miR-218 as a possible therapeutic for patients with bone metastatic breast cancer [76]. It is also known that lysyl oxidase (LOX), a crosslinker of collagen, is upregulated at metastatic sites (Figure 5b) [20,77]. Increases in LOX at the metastatic site increases ECM stiffness and facilitates cancer cell colonization [8]. Cox et al. has shown in tumor-bearing mice that there was an increase in bone loss and osteolytic lesion formation, which was LOX-dependent [78]. Cancer cells that were devoid of LOX that were injected into mice showed decreased osteolytic lesion formation [78]. This data suggests that LOX is used by cancer cells to change the biomechanical properties of bone remodeling, leading to excess bone degradation and the formation of osteolytic lesions [78].

### 3.2. Organic Bone Components: Non-Collagenous Proteins

There are other proteins besides collagen that are important for bone matrix structure and remodeling. Among the many non-collagenous proteins in the bone ECM, the top 5 most characterized proteins are Bone Sialoprotein (BSP), osteopontin (OPN), fibronectin (Fn), proteoglycans, and matrix metalloproteinases (MMPs) [4,5,79]. The first non-collagenous protein identified in bone was Bone Sialoprotein (BSP) [5]. Bone sialoprotein (BSP) is a protein of the small, integrin-binding ligand N-linked glycoprotein (SIBLING) family [80]. SIBLING family proteins, including BSP, have an RGD binding domain that mediates cell attachment and signaling, are secreted into the bone matrix during bone mineralization [81]. Gordon et al. found that BSP is also an important regulator of osteoblast differentiation and subsequent matrix mineralization [82]. Overexpression of BSP increased osteoblast-related gene expression of Runt-related transcription factor-2 (Runx2) and alkaline phosphatase enzyme activity, contributing to osteoblast differentiation [82]. In addition to BSP, another SIBLING family protein, OPN, is also an important organic component of the bone matrix [83]. OPN is expressed in a variety of tissues, including osteoblast progenitors and osteoblasts [80,83] is a critical component in cell-matrix interactions, bone resorption, and bone remodeling [83,84,85]. OPN regulates cell-matrix interactions through their RGD binding domain with integrins [80,86]. During biomineralization, OPN can bind directly to hydroxyapatite crystals, an inorganic component of the bone matrix, and inhibit mineralization [87]. OPN is also important for bone remodeling; bone cells secrete OPN during bone remodeling and can also increase OPN expression in response to mechanical stimuli [88,89]. It has been suggested that OPN also stimulates migration and bone resorption of osteoclasts through the cell surface adhesion receptor CD44 [90,91].

In bone metastatic cancers, such as breast and prostate cancers, bone matrix proteins, including BSP and OPN, have been implicated in the selective affinity of cancer cells to bone, through enhanced migration, invasion, and proliferation [92,93]. Carlinfante et al. identified that bone metastases from breast cancer patients had a higher expression of OPN compared to bone metastases from prostate cancer patients [93]. In contrast, bone metastases from breast cancer patients had a low expression of BSP compared to bone metastases from prostate cancer patients, suggesting that OPN and BSP expression are selective markers for the two types of metastases: osteolytic, more indicative of bone metastases from breast cancer patients and osteoblastic, more indicative of bone metastases from prostate cancer patients [93]. Another study demonstrated that inoculation of human breast cancer cells with BSP overexpression into athymic nude mice developed osteolytic bone metastases, whereas metastases that developed as a result of inoculation of human breast cancer cells with decreased expression of BSP did not develop osteolytic bone metastases. These results suggest that BSP may regulate osteolytic bone metastasis formation [94]. In addition, multiple studies have shown that OPN binding to cell surface adhesion receptor CD44 stimulates cancer cell migration, invasion, and metastasis [95,96,97]. Overall, phosphoproteins BSP and OPN are important regulators of cell-matrix interactions, bone mineralization, and bone remodeling, but are important mediators in tumor progression and metastasis [81,82,84,85,92].

In addition to the SIBLING family, there are other non-collagenous proteins, including proteoglycans, that are important in maintaining the bone matrix. Proteoglycans are one of the main classes of proteins found in the ECM and are important for formation and regulation of the bone matrix [10]. Proteoglycans are distinguished from other glycoproteins by the size and arrangement of the side sugar chains called glycosaminoglycans (GAGs) that are attached to a core protein [10]. GAGs are can be very large or very small [10]. Proteoglycans also regulate cell signaling by binding to proteins and (1) enhancing or decreasing the protein signal, (2) inhibiting the protein’s function by binding to it, or (3) by binding to the protein to inhibit it from being degraded [10]. Small, leucine-rich proteoglycans (SLRPs) are a subclass of proteoglycans are present during are found in mineralized bone matrix [98]. SLRPs participate in matrix organization binding to components of the bone matrix, such as collagen [4,98,99]. Bound and soluble SLRPs also regulate growth factor bioavailability and facilitate cell-matrix interactions by aiding in growth factor binding to receptors [99]. Decorin is a SLRP secreted by osteoblasts in the bone matrix [4,10,98,99]. Decorin regulates collagen fibril assembly and is essential for proper collagen fibril formation [10]. Decorin also participates in bioavailability of growth factors, such as the transforming growth factor β (TGF-β) [10,100]. TGF-β is a pleiotropic growth factor involved in many biological processes, including but not limited to, embryonic development, immune regulation, wound healing, and inflammation [101,102]. TGF-β was found to bind at the core protein and not the GAG chain of decorin [10,103]. When in the presence of collagen type I, decorin binds to TGF-β and sequesters it in the ECM [104]. Decorin has also been shown to bind TGF-β during bone remodeling and enhance its bioactivity [105]. These studies suggest that decorin may have a dual role in regulating growth factor bioactivity in the bone matrix. In addition to regulating growth factor bioactivity and matrix organization, decorin has anti-tumor properties in patients with bone metastases [100,106,107]. One pivotal study conducted by Nemani et al. investigated the interactions between bone cells and stromal cells and how decorin might be mediating this interaction when multiple myeloma cells are present [106]. The authors first looked at the expression of decorin in multiple myeloma cells and found that when compared to an osteosarcoma cell line that constitutively expressed decorin, multiple myeloma cells had no detectable amounts of decorin [106]. The authors next wanted to determine the expression of decorin in differentiating osteoblasts, bone marrow stromal cells (BMSCs) and osteoclasts [106]. Differentiating osteoblasts and BMSCs expressed high levels of decorin, especially during osteoblast differentiation, but when co-cultured with multiple myeloma cells, decorin expression decreased, which is thought to be due to decreased osteoblast differentiation [106]. Osteoclasts from multiple myeloma patients expressed decreased amount of decorin, but exogenously adding decorin to a differentiating culture of precursor osteoclasts yielded a decrease in the number of TRAP positive osteoclasts, suggesting that decorin inhibits osteoclast differentiation [106]. Overall, this study demonstrates that decorin has anti-tumor effects that modulate the tumor microenvironment indirectly [106].

Fibronectin (Fn), a matrix glycoprotein, mediates many cellular interactions within the bone matrix, including but not limited to, cellular adhesion, migration, and differentiation (Figure 5a) [108]. Fn can be divided into two sub forms: plasma Fn, which is mainly produced by hepatocytes in the liver and is soluble; and cellular Fn, which can be produced by different cell types and tissues, and is relatively insoluble [108]. Cellular Fn is cell-type-specific, meaning depending on the tissue type, the splicing of Fn may vary [108]. Each variant could have different adhesion, ligand binding, or solubility properties that may be tissue dependent [108]. In the bone, Fn is secreted mainly by fibroblasts, but in the bone matrix, osteoblasts are the main producers of Fn [10,109]. Fn is also known to regulate osteoblast differentiation [109,110]. Faia-Torres et al. has shown that having a low Fn-density matrix was able to promote the differentiation of human MSCs to an osteogenic lineage determined by the expression of alkaline phosphatase and collagen type I staining [111]. In addition to osteoblast differentiation, Fn has a collagen binding domain that serves as a scaffold for collagen fibril formation [108,112,113]. There is controversary as to whether denatured collagen or native collagen binds more effectively in this region, but there is evidence supporting that both denatured and native collagen can bind to the collagen binding domain of Fn [108,114]. The deposition of Fn into the bone ECM by cells of the bone microenvironment is a tightly regulated process [108]. Fn has been shown to be manipulated by other cells in the bone microenvironment, such as cancer associated stromal cells during tumorigenesis [115,116,117]. Studies have demonstrated that tumor cells signal to the surrounding tumor stroma to produce Fn, since cancer cells cannot produce their own Fn matrix [116,117]. The cancer-initiated Fn matrix is highly unorganized and composed of thick, dense fibrils (Figure 5b) [116,117]. The remodeling of the bone matrix by tumor stroma cause further mechanical and structural changes, which is mediated by MMPs [117].

MMPs, are proteolytic enzymes mainly responsible for matrix degradation, including the bone matrix, as well as protein cleavage (Figure 5a) [79]. MMPs can cleave precursor proteins, such as pro-MMP precursors and activate them [1]. Most MMPs can exist as secreted proteins or membrane-bound proteins, but all target a wide range of ECM molecules [7,118]. For example, MMP-3 and MMP-10 selectively target proteoglycans and fibronectin, whereas MMP-8 and MMP-14 selectively target collagen type-1 and MMP-9 degrades denatured collagen [1,79]. It is known that various subtypes of bone cells produce different MMPs; osteoblasts produce MMP-1, MMP-2, MMP-13, and MMP-14, whereas it has been suggested that osteoclasts solely produce MMP-9 [119,120,121,122]. MMP-9 is activated by cleavage of the pro-domain by various MMPs, such as MMP-2 [123,124], MMP-3 [124], and MMP-13 [125]. MMP-9 is important for chondrocyte apoptosis during endochondral ossification [126]. MMP-9 is also highly expressed during fracture healing, whereby it aids in degradation and stabilization of the bone matrix [7]. MMP-2, MMP-13, and MMP-14 have multiple functions during osteogenic differentiation, including acting as a major degrader of collagen type I during pre-osteoblast differentiation [127,128] and early ossification of bone [7]. Tauro et al. demonstrated that increases in MMP-2, MMP-3, MMP-13, and MMP-9 expression correlated with increases in bone matrix degradation [129]. MMPs have also been shown to be important in cancer progression, whereby MMPs are upregulated, resulting in excessive matrix degradation and remodeling (Figure 5b) [1,118]. Therefore, targeting MMPs during cancer progression may decrease tumor outgrowth. Perentes et al. demonstrated that downregulating MMP-14 in breast cancer cells reduced blood vessel invasion and spontaneous metastasis in a triple negative breast cancer model [130]. Tauro et al. has shown using a MMP-2 inhibitor that specifically targets bone, tumor-associated bone destruction and tumor growth was reduced in vivo [129]. In addition, they found that the MMP-2 inhibitor targeted breast cancer cells and osteoclasts, but not osteoblasts in vitro, suggesting decreased bone destruction [129]. Bruni-Cardoso et al. studied the effects of stromal-derived MMP-9 on the progression of prostate cancer in bone and found MMP-9 was able to induce prostate cancer tumor progression without contributing to changes in bone composition [131].

### 3.3. Inorganic Bone Components

Inorganic bone matrix is a rich source of minerals, including calcium and phosphate, which are released during bone resorption [5]. The inorganic bone matrix is mainly composed of hydroxyapatite crystals, which allow for mineral exchange in bone (Figure 5) [5,66]. Extensive studies have shown that collagen deposition can initiate and orientate hydroxyapatite crystal formation, which are both vital for bone matrix mineralization [68,132]. Nakano et al. identified that rabbit ulna and skull bone varied in their structure of hydroxyapatite orientation via a suggesting that apatite crystallization is related to stress distributions in bone [133,134]. Furthermore, Sekita et al. has shown that the abnormal arrangement of apatite crystals, in conjunction with collagen fibers, impairs bone mechanical function and disrupts osteoblast alignment [66]. To study bone mechanical function, the authors inoculated mouse femurs with or without prostate cancer cells and analyzed collagen and hydroxyapatite orientation and bone density [66]. The authors found that mice inoculated with prostate cancer cells had a non-directional bone patterning, compared to unilateral bone formation in mice not inoculated with prostate cancer cells, which was further determine to be due to the abnormal alignment of collagen and hydroxyapatite crystals [66]. The abnormal alignment of apatite crystals and collagen fibers was further found to disrupt osteoblast alignment during both breast and prostate cancer bone metastatic progression [66,135].

## 4. Bone Metastatic Cancers

Primary bone cancer, such as osteosarcoma, is rare [136], but cancers that metastasize to bone are quite common [137]. Bone is the third leading site of cancer metastases, behind lung and liver [137]. Bone is a preferential site of metastasis because of its high metabolic state due to constant bone turnover, releasing growth and survival signals into the bone microenvironment which may stimulate cancer cell survival [60]. Furthermore, bone contains vascular sinusoids, which are areas of sluggish blood flow [10,11]. Metastatic cancer cells take advantage of this sluggish blood flow and primarily enter the bone via the vascular sinusoids [138,139]. Bone metastatic lesions most commonly present in patients previously diagnosed with prostate cancer, breast cancer, or multiple myeloma [140,141]. Bone metastases are usually classified as osteolytic or osteoblastic, although some metastases can be mixed [137]. Osteoblastic metastases are characterized by increased bone deposition, whereas osteolytic lesions are characterized by excessive bone resorption [137].

### 4.1. Prostate Cancer

Patients with bone metastatic prostate cancer can present with either osteoblastic, osteolytic, or mixed metastases [142], but most commonly present with osteoblastic lesions [140,143,144]. Schneider et al. demonstrated that bone turnover induced prostate cancer cell localization to the long bones of athymic mice [145]. Athymic mice were treated with recombinant parathyroid hormone (PTH), a well-known stimulator of bone turnover, inoculated mice with prostate cancer cells, and found that mice treated with PTH had increased bone formation adjacent to tumor regions compared to control mice [145]. This data suggests that cancer cells localize to more active sites of bone, activating bone turnover and stimulating tumor colonization and growth [145].

The bone matrix houses a plethora of cytokines and growth factors, including transforming growth factor beta (TGF-β) [2,51]. TGF-β is a known potent mitogen for osteoblast formation [4], where osteoblasts produce increased amounts of TGF-β in sclerotic bone compared to normal bone [146]. Therefore, mechanical stimuli or bone remodeling release these factors into the bone microenvironment, whereby cancer cells utilize these factors to stimulate cancer growth [1,2,8,147]. Meng et al. used a knockout mouse model of TGF-β receptor 2 (TGFBR2) in osteoblasts and a knockout mouse model of TGFBR2 in osteoclasts to determine the effects of TGF-β signaling in prostate cancer bone metastases [148]. After intratibial or intracardiac inoculation of prostate cancer cells, knockout of TGFBR2 in osteoblasts promoted bone lesion formation and knockout of TGFBR2 in osteoclasts inhibited bone lesion formation [148] Using a cytokine array, the authors identified basic fibroblast growth factor (bFGF) as the most upregulated growth factor in tibias from in osteoblasts from TGFBR2 knockout mice inoculated with prostate cancer cells, and was further found in osteoblasts to be the mediator of the prostate cancer growth [148]. This data suggest loss of TGF-β signaling in osteoblasts has an a metastasis-promoting effect through bFGF in a prostate cancer bone metastasis model [148]. 

It has also been shown that osteonectin, a collagen binding bone matrix protein, is upregulated in prostate cancer bone metastases and stimulates the invasion and migration of prostate cancer cells [149], however other studies have shown that osteonectin-null mice had accelerated cancer progression, invasion and metastases [150,151]. Because of the discrepancy in the literature, Kapinas et al. wanted to devise the role of osteonectin in prostate cancer bone metastases using mineralized matrices produced by osteonectin-null and wild-type prostate cancer cells [152]. The authors found that osteonectin-null matrices had a non-directional, thin matrix compared the directional and collagen-thick wild type matrix [152]. The authors further found that prostate cancer cells grown on the wild type matrices exhibited decreased cell proliferation and increased cell spreading, suggesting that osteonectin may play a role in inhibiting prostate cancer growth [152]. 

It is known that metastatic prostate cancer cells can attach to osteoblasts in the bone microenvironment to facilitate tumor progression [153]. Kimura et al. has demonstrated that physical contact between prostate cancer cells and osteoblasts disrupts osteoblast alignment on a bone matrix, further contributing to remodeling of bone microstructure [135]. Similarly, Seikta et al. demonstrated that mouse femurs inoculated with prostate cancer cells induced a non-directional bone forming pattern, where alignment of collagen and apatite crystals and bone toughness was decreased [66]. Prostate cancer bone metastasis may also contribute to changes in the bone matrix. Particularly, Sottnik et al. has shown that tumor-generated pressure in mouse tibias modified the bone microenvironment and induced the growth of prostate cancer cells [29]. Further investigation revealed that the exerted pressure induced osteocyte expression, and through bone matrix remodeling effector C-C motif chemokine ligand 5 (CCL5), and MMPs, promoted the growth of prostate cancer bone metastases [29]. 

### 4.2. Breast Cancer

Breast cancer also preferentially metastasizes to bone [62,141]. Bone metastatic breast cancer patients can have osteoblastic, osteolytic, or mixed lesions [154], but patients predominantly present with osteolytic lesions [155], whereby osteoclasts are overactive [156,157]. When breast cancer cells enter bone, bone homeostasis is disrupted and the balance is shifted to favor bone resorption and remodeling of the bone matrix [4]. The formation of osteolytic lesions occurs when communication between osteoblasts and osteoclasts is disrupted [27]. It is well established that TGF-β induces secretion of parathyroid hormone-related protein (PTHrP) from breast cancer cells and increases the production of RANKL from osteoblasts to stimulate osteoclast formation and activation [27,158]. Osteoclasts then resorb bone, releasing cytokines and growth factors, including TGF-β, which cancer cells can use to produce more PTHrP [19,62]. This process is known as the ‘vicious cycle’ of bone degradation, which also contributes to increased bone matrix remodeling [19,27,60]. In addition to bone resorption, patients exhibiting primary and metastatic breast cancer tumors with high desmoplasia or increased fibrosis, have increased expression of stromal cell collagen and fibronectin and MMPs, corresponding with increased bone extracellular matrix remodeling and poor patient outcome [59,159,160]. For example, in breast cancer metastasis, MMP-9 is associated with degradation of bone matrix through the activation of p38, a mitogen activated kinase [161], or by cleavage with cathepsin K, a proteinase responsible for matrix degradation [162]. While there is evidence that ties specific ECM components to breast cancer metastases, there is much less known about how mechanical cues facilitate tumor progression [163]. During breast cancer metastasis, ECM remodeling relates closely to bone resorption [4] and previous data has indicated that alterations in the tumor microenvironment cause increases in pressure and compression, leading to ECM stiffening and cell contractility [164,165]. Page et al. has shown that increased rigidity in mineralized bone matrix stimulates tumor cells to take on a bone destructive phenotype by altering the expression of genes associated with bone destruction [166]. They showed that tumor-produced gene expression of Gli2 and PTHrP, two genes that regulate bone remodeling, were significantly increased when breast cancer cells were cultured on a rigid 2D matrix compared to a less stiff 2D matrix, suggesting that rigidity of matrices can change the alter expression of genes involved in bone remodeling [166]. The authors identified integrin β3 (Iβ3) and TGRFR2, two growth factor receptors regulated by TGF-β signaling, as regulators of Gli2 and PTHrP, whereby both receptors co-localized on rigid matrices compared to less rigid matrices, suggesting that a rigid matrix can change gene expression and bone destruction through mechanosignaling [166]. 

### 4.3. Multiple Myeloma

Multiple myeloma (MM) is also known to cause alterations in the bone microenvironment, leading to osteolytic bone lesions [167]. Multiple myeloma is a plasma cell cancer that homes to the bone marrow, causing severe skeletal complications, hypercalcemia, and fatigue [168]. Changes also occur in the bone microenvironment, including increased angiogenesis and interactions between bone marrow stromal cells (BMSCs) and myeloma cells, contributing to tumor progression [169]. A study by Wu et al. demonstrated that BMSCs from MM patients were stiffer than BMSCs from normal volunteers as measured by the atomic force microscope (AFM), suggesting that microenvironmental changes can regulate cell behavior, which may contribute to disease progression [170]. Furthermore, CD138^-^ myeloma cells, but not CD138^+^ myeloma cells, were responsible for regulating stiffness of BMSCs [170]. CD138^-^ myeloma cells were identified clonal subpopulation of multiple myeloma cancer stem cells that have continuous self-renewal property and were found to be in the bone marrow of multiple myeloma patients associated with poor survival [171,172]. Studies have identified this CD138^-^ stem cell population express stromal cell-derived factor-1 (SDF-1), which regulates homing of multiple myeloma cells to the bone marrow, and its receptor C-X-C motif chemokine receptor 4 (CXCR4) [170,173]. The Protein Kinase B (also known as AKT) signaling pathway was previously found to mediate prostate cancer cell migration and invasion via the SDF-1/CXC4 axis [174]. Wu et al. determined that CD138^-^ multiple myeloma cells regulate BMSC stiffness though the SDF-1/CXC4/AKT signaling pathway in the bone microenvironment [170]. These studies demonstrate the microenvironmental changes, such as matrix rigidity, can affect cell behavior and change the expression of certain genes or active pathways that are associated with tumor progression.

In addition, another study by Vallet et al. has shown that microenvironmental C-C motif chemokine 3 (CCL3), and its receptors C-C chemokine receptor type 1 (CCR1) and C-C chemokine receptor type 5 (CCR5), are important in promoting osteolytic lesion formation, through regulation of osteoclast differentiation, and tumor progression in multiple myeloma patients [175]. CCL3 is proinflammatory chemokine modulates osteoclast differentiation by binding to its receptors CCR1 and CCR5 activating the AKT and extracellular signaling regulated kinase (ERK) signaling pathway [175]. CCL3 has also been shown to promote multiple myeloma cell migration and survival in the bone microenvironment [176]. This study found CCL3 is responsible for the inhibition of osteoblast function through the activation of ERK, subsequent downregulation of osterix, an osteogenic transcription factor, and expression of osteocalcin, a osteoblast differentiation marker [175]. Furthermore, inhibition of CCR1 decreased ERK activation and increased expression of osterix and osteocalcin, when in the presence of CCL3, suggesting CCL3 is an important regulator of osteoblast and osteoclast function, leading to the uncoupling of osteoblast and osteoclast homeostasis in multiple myeloma [175]. Another study also found osteoblast function was mediated by CCL3 in multiple myeloma in which multiple myeloma cells decreased osteoblast-induced decorin secretion [106]. Decorin was produced by osteoblasts, but not by multiple myeloma cells, suggesting that decorin is an inhibitory molecule for multiple myeloma survival in the bone microenvironment [106]. These studies demonstrate that microenvironmental signals and interactions with surrounding cells are important in initiating multiple myeloma tumor growth. 

### 4.4. Lung Cancer

Lung cancer metastasizes to bone approximately 34.3% of the time, making the skeleton a preferential site of metastasis [177]. Interactions with the bone stroma appear to drive lung cancer homing and colonization, whereby factors expressed by bone marrow stromal cells, osteoblasts, and osteoclasts, such as platelet derived growth factor receptor beta, promote metastatic lung cancer engraftment in bone [178]. In another example, Vicent et al. determined that bone resorption as driven by TGF-beta, anchorage-dependent factors including melanoma cell adhesion molecule (MCAM) and Sushi domain-containing protein 5 (SUSD5), and protein kinase D3 (PRKD3), a protein kinase that modulates the activity of matrix metalloproteinases during ECM remodeling [179], all promoted increased bone metastatic lung cancer colonization and growth [180]. In one final example, Tang et al. observed a role for the stromal-derived factor-1 (SDF-1) CXCR4 axis in the chemoattraction of lung cancer cells to bone [181]. The authors isolated mRNA and protein from the highly aggressive lung cancer cell line A549 and compared it to mRNA and protein isolated from lung cancer cell lines that are less aggressive, including H928 and H1299 cells. The authors determined that the CXCR4 receptor, which binds with high affinity to SDF-1, was highly expressed in the aggressive A549 cells when compared to the less aggressive H928 or H1299 cells. SDF-1/ CXCR4 interaction was directly responsible for the chemoattraction of lung cancer cells in Boyden chamber assays. Further analysis showed that lung cancer cells’ interaction with SDF-1 mediated the upregulation of MMP9 expression which further increased lung cancer cell chemoinvasion to bone [181]. Thus, these studies suggest that factors involved in remodeling of the bone matrix promote lung cancer cell homing to bone.

The available literature suggests that two of the more common types of lung cancer, non-small cell lung cancer (NSCLC) and small cell lung cancer (SCLC), metastasize to bone and present mainly with osteolytic bone metastases of the spine and ribs. These lesions can also be mixed osteoblastic and osteolytic [182,183,184]. Interestingly many bone turnover markers, including bone sialoprotein (BSP), collagen type I, and osteopontin (OPN) can be used as biomarkers for the diagnosis, prognosis, and evaluation of lung cancer bone metastases including that of NSCLC and SCLC [82,185,186,187]. In particular, He and colleagues found that NSCLC patients with bone metastases had higher BSP serum levels compared with NSCLC patients without bone metastases [188]. Furthermore, meta-analysis data correlated type I collagen with the progression of bone metastases in lung cancer patients [189,190,191,192]. In another study, Valencia and colleagues identified that knockdown of the discoidin domain receptor-1 (DDR1), a receptor for type I collagen, in the lung cancer cells reduced bone metastatic burden as measured by tumor burden and osteolytic lesion formation in a mouse model of lung cancer bone metastasis [193]. The authors further found that lung cancer cells with knockdown of DDR1 exhibited significantly decreased bone tumor burden [193]. 

OPN, as a biomarker, is increased in patients with NSCLC and is associated with an aggressive lung cancer phenotype [194]. OPN promotes lung cancer cell migration by driving lung cancer cell epithelial-to-mesenchymal transition (EMT) [195], as well as lung cancer cell interactions with integrins [196]. Integrins are transmembrane receptors that mediate cell-matrix and cell-cell adhesion [10]. Roman et al. found that fibronectin interacts with integrin α1β5 through receptor-mediated signaling, which is important for lung cancer metastasis to bone [197]. To test the role of fibronectin and integrin α1β5 interaction on metastatic potential of lung cancer bone metastasis in vivo, Roman and colleagues silenced the α5 subunit in lung carcinoma cells [197]. When α5-silenced lung carcinoma cells were injected into C57BL/6 mice, there was a decrease in bone metastatic burden compared to control wild-type or α2-silenced carcinoma cells [197]. Thus, these studies as a whole demonstrate that bone turnover markers and matrix proteins are important predictors for progression of bone metastatic lung cancer. 

## 5. Current Therapies Targeting Bone Metastases

Bone is a common site of metastasis and patients with bone metastasis report the worst quality of life of all sites of metastasis. This is due to debilitating skeletal related events associated with bone metastases, including bone pain, fractures, and hypercalcemia. As previously described, in many cases of bone metastasis, osteolytic lesions are common whereby osteoclasts are overstimulated to resorb bone and osteoblasts fall short in building new bone, resulting in net bone loss [19,59,61]. To combat excess bone degradation by osteoclasts, bisphosphonates, which are inorganic pyrophosphates, are commonly used in the clinic [17,198]. Bisphosphonates bind to exposed bone mineral produced by resorbing osteoclasts, resulting in high concentration of the drug in the resorptive pit [137]. The bisphosphonates are then internalized by osteoclasts which cause disruption of the chemical process of bone resorption, ultimately result in osteoclast apoptosis [199,200,201]. Bisphosphonates are well tolerated by patients, with mild to moderate flu-like symptoms being the most common side effect [201]. Bisphosphonates are used to help with the symptoms of bone metastases, such as osteolytic lesion formation, but they are not curative [137].

Current Food and Drug Administration (FDA)-approved bisphosphonates used in the clinic for bone metastases are categorized as either first, second, or third generation bisphosphonates [137,198]. First generation bisphosphonates include clodronate. Examples of second-generation bisphosphonates are pamidronate and alendronate. And, examples of third generation bisphosphonates include ibandronate and zoledronic acid [202,203].

First generation bisphosphonates are non-nitrogen containing, so disruption of osteoclasts occurs via cellular metabolism, leading to osteoclast apoptosis [204]. The first generation bisphosphonate clodronate was originally approved in Europe in 1992 for management of skeletal related events, including osteolytic lesions, bone pain, and hypercalcemia associated with breast cancer or multiple myeloma [202]. On the other hand, second and third generation bisphosphonates are different from first generation bisphosphonates because they contain a nitrogen-containing side group [202]. Second and third generation bisphosphonates are more potent than the first-generation bisphosphonates because they impair intracellular osteoclast signaling by inhibiting farnesyl diphosphate synthase (FPP) pathway [198,202,205,206]. The FPP pathway inhibits osteoclast activity and induces osteoclast apoptosis [205,206]. The second generation bisphosphonate pamidronate, commonly known as Aredia, was first approved for clinical use in the US in 1996 for the treatment of osteolytic metastasis in breast cancer [207]. The second-generation bisphosphonate alendronate, commonly called Fosamax, is associated with reduced bone metastases in post-menopausal women. In a study of 297 osteoporotic women carried out by Rouach and colleagues, women who were treated with alendronate exhibited a reduced risk of developing bone metastatic breast cancer when compared to patients who were not treated with alendronate [208]. Finally, third generation bisphosphonates differ from second generation bisphosphonates by the location of the nitrogen group [209]. The nitrogen group is on a different side chain, which allows for a more potent reaction of FPP, thereby leading to an increase of osteoclast inhibition [209]. The third generation bisphosphonate zoledronic acid (ZA), commonly known as Zometa or Reclast, was first approved in 2001 for the treatment of skeletal complications related to bone metastases [202]. ZA has been shown to be more effective for the management of skeletal related events and skeletal complications in breast and prostate cancer compared to other bisphosphonates [210,211,212]. At the present, ZA is the most effective bisphosphonate clinically available and is currently the standard of care used to treat patients with bone metastases [213].

In addition to bisphosphonates, other therapies, such as RANKL monoclonal antibodies, are being used in the clinic to treat osteoclast resorption [202]. The first and only RANKL-monoclonal antibody, denosumab, was approved in 2010 for the management of bone metastases and for the prevention of bone pain, fractures, or hypercalcemia [202,204]. Denosumab functions by binding to soluble and membrane-bound RANKL with high affinity [204,214]. This inhibits RANKL from binding to the RANK receptor on osteoclasts, decreasing osteoclast formation and activity [204,214]. Denosumab is given as a subcutaneous injection, compared to bisphosphonates, which are given intravenously [202,214]. Subcutaneous injections greatly increase the convenience and attainability of administration and treatment of the drug [200,203]. 

There are currently no drugs available to directly stimulate the activity of osteoblasts and thus promote bone formation, but romosozumab, a sclerostin inhibitor and commonly known as Evenity, is used to increase bone formation [215,216]. Romosozumab works by inhibiting the actions of sclerostin, an inhibitor of bone formation. Under normal circumstances, when sclerostin, a secreted glycoprotein, binds to its receptor, low-density lipoprotein receptor-related protein 5/6 (LRP5/6) and co-receptor Frizzled, on osteoblasts β-catenin phosphorylation is inhibited resulting in β-catenin degradation, and ultimately inhibiting osteoblast bone formation [215,217]. Romosozumab blocks sclerostin from binding to LRP5/6 and Frizzled, thus promoting bone formation [215,217]. Sclerostin also stimulates RANKL secretion to induce osteoclastogenesis [218]. Sclerostin inhibitors, including romosozumab, bind to sclerostin and inhibit it from binding to its receptors, resulting in continuous bone formation and decreased osteoclastogenesis [217,219,220]. 

## 6. Models to Study the Bone and Bone Matrix

**Figure 6:**
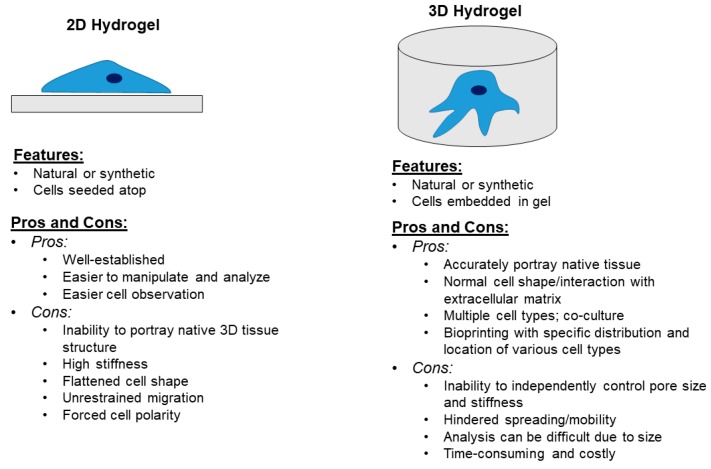
Hydrogels as models of the bone matrix. Hydrogels are unique model systems that can be used to determine interactions between cells and the bone matrix. This image shows cellular comparisons between 2D and 3D hydrogels. Cells are cultured atop a 2D hydrogel coated with a matrix, such as collagen, whereas cells are embedded in 3D hydrogels.

Biophysical properties of bone and the bone matrix are important determinants of cell behavior [4,8]. The cell-matrix interactions in bone can effect cell migration, proliferation, survival, and remodeling [8,221], however studying them can be difficult. One limitation to studying bone and bone matrix remodeling is the ability to recapitulate a bone-mimetic microenvironment in vitro [222,223,224]. Bone is a complex structure and without the use of animal models, recapitulating a bone microenvironment in a laboratory setting can be challenging [222,224]. Therefore, the development of unique model systems to determine interactions between cells and the bone matrix is essential. One alternative to this quandary is to use 2D or 3D hydrogels (Figure 6) [222,223,224]. Most commonly used for in vitro work are 2D hydrogels, which can be either natural or synthetic [222]. Both natural and synthetic hydrogels have advantages and disadvantages. Natural hydrogels, such as collagen, are obtained from organisms, such as rat tail tendon, and do not have to be significantly modified, whereas synthetic hydrogels, such as polyacrylamide, are readily available from laboratory supply companies, but need to be adjusted to fit a specific range of substrate mechanics [135,166,222]. A major disadvantage to 2D hydrogels is the inability to accurately portray 3D structure of native tissues, and subsequently the biophysical properties, such as elastic modulus and tissue stiffness [223]. 

When choosing a hydrogel, it is important to determine how the cells will adhere to a natural or synthetic hydrogel and how this may effect stability and biophysical properties [222]. A cell can behave differently on different types of material, including natural or synthetic hydrogels, thus it is important to determine the context of the environment. For example, a natural hydrogel should be picked for cells that normally grow on a collagen matrix because collagen is a natural hydrogel [222]. Cells can be seeded atop a hydrogel (2D) or be embedded in a hydrogel (3D) [222,224]. Use of a 2D versus a 3D hydrogel, or a natural material versus a synthetic material, will depend upon the experimental design and what output is most important. For example, the best 3D hydrogels to use if wanting to accurately portray the structure of the bone matrix, would be a natural 3D hydrogel consisting of collagen because this structure would accurately portray the composition, density, and mechanotransduction properties of the tissue [223].

3D hydrogels best mimic native tissue, which can lead to more realistic cellular responses, such as cell physiology and mechanotransduction [222,223,224]. Drawbacks to using 3D hydrogels are that cells will have hindered spreading or mobility because of being surrounded by matrix [224], as well as the inability to independently control pore size and stiffness [223]. To combat these limitations, Cassereau et al. developed a 3D tension bioreactor that allows for constant mechanical tuning of a native collagen I hydrogel stiffness, without any alterations to the structure, composition, or pore size of the gel [223]. The group wanted to determine the impact of ECM stiffness on tumor progression, independent of structural changes to the ECM [223]. They found that increasing ECM rigidity, by increasing collagen concentrations, was able to induce tumor cell invasion [223]. 

Lastly, scaffolds can also be used as an alternative approach to study the bone microenvironment. Biomimetic scaffolds are 3D structures that are usually made with a synthetic polymer specific to the type of environment best suited to the cell type in use. For example, Seib et al. used a highly porous silk scaffold that was biocompatible to bone and had bone morphogenetic protein 2 (BMP2) properties [225]. In this way, scaffolds were used to model the microenvironment as accurately as possible without using an in vivo approach [225,226]. In addition, cells can also be seeded onto the scaffold and growth in vitro, and then implanted into animals, such as mice [226].

## 7. Concluding Remarks, Challenges, and Future Perspectives

The bone is a fertile soil for metastatic cancer cells, and the bone components, including the bone matrix, are essential in facilitating cancer growth. The bone matrix has emerged as a central player in primary and metastatic cancer, which allows the ECM to be an active participant through different stages of disease progression. The dynamic nature of the matrix makes it a necessary target for deregulation by cancer cells. It is becoming increasingly evident that bone matrix proteins, including organic component collagen type I and decorin, are being remodeled and manipulated to govern cancer growth in bone.

Now, researchers are starting to realize the importance of the bone during disease progression, especially in the case of therapeutic intervention. It will be important to determine which components of the bone ECM are most critical in facilitating disease progression and how these changes may affect cancer growth. One challenge that researchers are currently facing is the lack of model systems to study bone metastatic cancers. Understanding the mechanisms behind these events will lead to a better understanding of what factors are altered during bone matrix remodeling in bone metastatic cancer, and how these changes contribute to disease progression.
